# Foreword

**DOI:** 10.3402/gha.v6i0.20433

**Published:** 2013-02-25

**Authors:** Nguyen Duc Hinh

**Affiliations:** President of the Hanoi Medical University, Hanoi, Vietnam

I am very pleased to introduce the *Global Health Action* cluster of scientific papers on ‘Public health in Vietnam: here's the data, where's the action?’ by the Hanoi Medical University, Hanoi, Vietnam, and Epidemiology and Global Health, Umeå University, Umeå, Sweden. The objectives of the supplement are to: 1) provide scientific evidence for policy changes and interventions; 2) share research findings on various critical public health issues from Vietnam with the rest of the world; and 3) improve the capacity of Vietnamese scientists for writing international scientific articles.

This cluster, consisting of 15 papers, not only provides up-to-date scientific evidence on the situation of health status and health care in Vietnam but also suggests recommendations for policy and public health actions to improve these situations. The cluster, one of a few clusters of peer-reviewed scientific papers from Vietnam, demonstrates that international scientific publications are both necessary for and possible from Vietnam.

I wish to thank the Swedish International Development Cooperation (Sida), through the Swedish Embassy in Hanoi, for funding this supplement. I acknowledge Dr. Lucia D'Ambruoso, Prof. Stig Wall, Assoc. Prof. Nawi Ng, *Global Health Action*, Sweden, Prof. Ta Thanh Van and Assoc. Prof. Hoang Van Minh, Assoc. Prof. Kim Bao Giang, Hanoi Medical University, Vietnam for their editorial support and coordination. I am grateful to Prof. Lars Weinehall from Epidemiology and Global Health, Umeå University, for his technical and managerial contribution to the preparation of this cluster. I deeply appreciate the continued efforts by the authors, mentors and reviewers who have made these papers possible.

I sincerely hope that this cluster of papers will inspire Vietnamese scientists to do more research for international peer-reviewed publications. I also expect that the results published in this supplement will result in proactive public health actions to respond to public health issues in Vietnam and elsewhere!

*Nguyen Duc Hinh* President of the Hanoi Medical University Hanoi, Vietnam

